# Instructor-learner body coupling reflects instruction and learning

**DOI:** 10.1038/s41539-022-00131-0

**Published:** 2022-06-28

**Authors:** Yafeng Pan, Suzanne Dikker, Yi Zhu, Cuirong Yang, Yi Hu, Pavel Goldstein

**Affiliations:** 1 Department of Psychology and Behavioral Sciences, Zhejiang University, Hangzhou, China; 2 Shanghai Key Laboratory of Mental Health and Psychological Crisis Intervention, Institute of Brain and Education Innovation, School of Psychology and Cognitive Science, East China Normal University, Shanghai, China; 3 NYU-Max Planck Center for Language, Music and Emotion, New York City, NY USA; 4 Department of Clinical Psychology, Vrije Universiteit Amsterdam, Amsterdam, The Netherlands; 5 Department of Psychology, Suzhou University of Science and Technology, Suzhou, China; 6 Shanghai Center for Brain Science and Brain-Inspired Technology, Shanghai, China; 7 Integrative Pain (iPain) Laboratory, School of Public Health, University of Haifa, Haifa, Israel

**Keywords:** Human behaviour, Education

## Abstract

It is widely accepted that nonverbal communication is crucial for learning, but the exact functions of interpersonal coordination between instructors and learners remain unclear. Specifically, it is unknown what role instructional approaches play in the coupling of physical motion between instructors and learners, and crucially, how such instruction-mediated Body-to-Body Coupling (BtBC) might affect learning. We used a video-based, computer-vision Motion Energy Analysis (MEA) to quantify BtBC between learners and instructors who used two different instructional approaches to teach psychological concepts. BtBC was significantly greater when the instructor employed a scaffolding approach than when an explanation approach was used. The importance of the instructional approach was further underscored by the fact that an increase in motion in the instructor was associated with boosted BtBC, but only during scaffolding; no such relationship between the instructor movements and BtBC was found during explanation interactions. Finally, leveraging machine learning approaches (i.e., support vector and logistic regression models), we demonstrated that both learning outcome and instructional approaches could be decoded based on BtBC. Collectively, these results show that the real-time interaction of teaching and learning bodies is important for learning and that the instructional approach matters, with possible implications for both in-person and online learning.

## Introduction

Humans, like other species, have demonstrated exceptional abilities to learn about values, skills, and knowledge through dyadic interactions with conspecifics. Such instructor-learner interaction during information transmission is ubiquitous in our daily lives, from an early age on, and have been shown to be supported through a variety of mechanisms; examples include selective attention to others^1,2^, observation and imitation^3,4^, as well as prediction error or theory of mind^5,6^. Yet, existing studies have mainly focused on single individuals, leaving the behavioral dependencies between individuals less explored.

Filling this gap, converging strands of evidence have demonstrated that interpersonal behavioral coupling serves as a mechanism for human social behaviors and affiliation bonding, including prosociality^7^, music making^8^, relationship quality^9^, and collaborative problem-solving^10^. Previous studies have also identified interpersonal movement coordination between learners and instructors^11,12^, but the relationship between synchronized body movement, learning, and instruction (e.g., academic performance, instructional approaches) is largely unknown, despite the fact that numerous studies have demonstrated a relationship between the quality of learning interactions and learning satisfaction. Positive instructor-learner interactions are known to contribute to students’ learning engagement^13^, comfort^14^, school satisfaction^15^, and eventually to academic performance^16^. Conversely, insufficient learning interactions and/or poor interaction quality are associated with low levels of emotional engagement^17^ and negative test scores^18^. These findings strongly suggest that a better understanding of the real-time interactions between instructor and learner may support teaching and learning, resulting in more effective and enjoyable approaches.

**Fig. 1:**
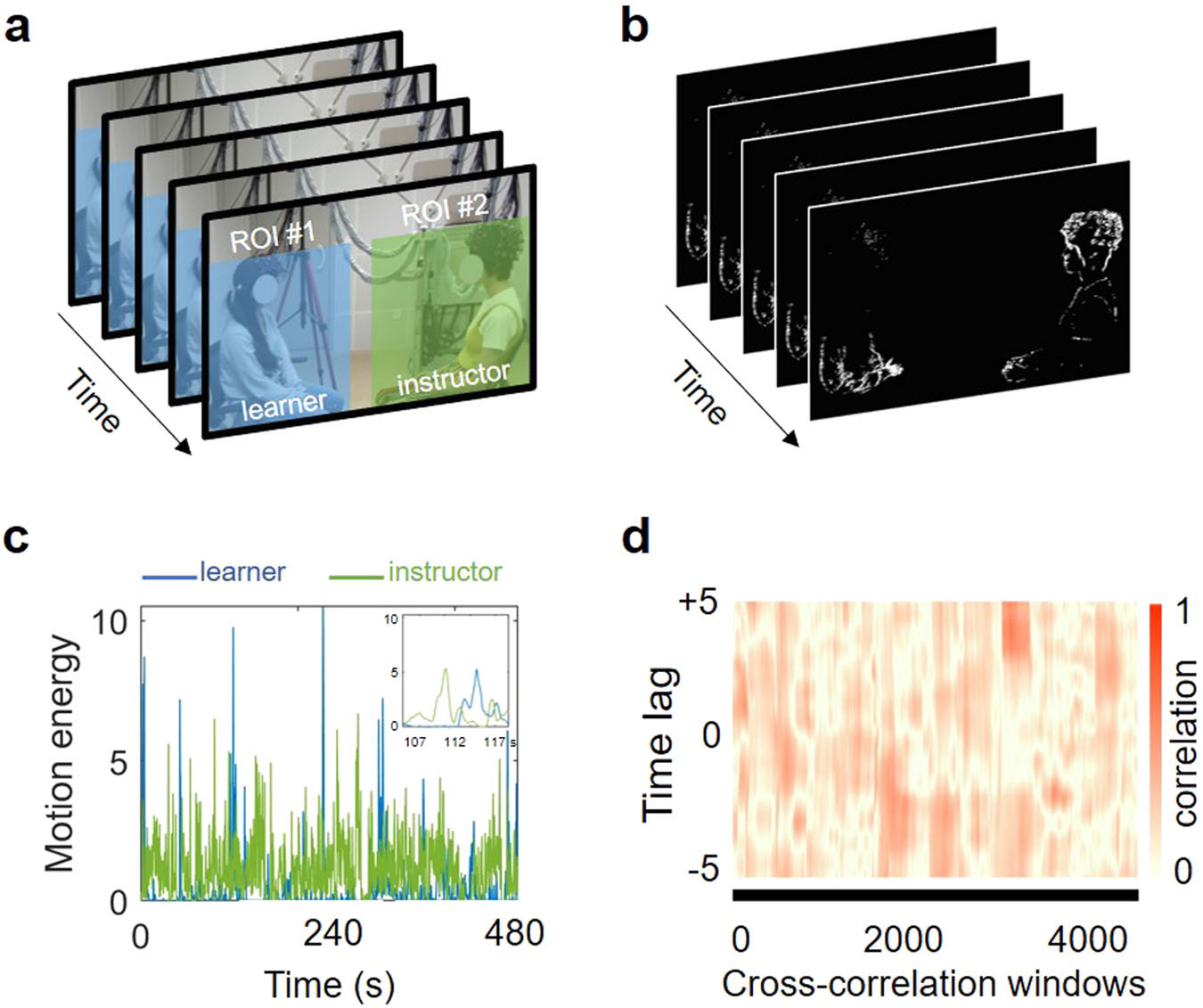
Motion energy analysis. **a** Regions of interest (ROIs) in instructor-learner dyads for the video-based motion energy analysis. ROI #1 and ROI #2 cover the full body of the instructor and that of the learner, respectively. **b** Corresponding frame-differenced video (blue line = learner, green line = instructor). **c** Preprocessed motion energy within each ROI as a function of the video frame. The right top box indicates a representative selected chunk of the time course. **d** Body-to-Body Coupling (BtBC) between the learner (ROI #1) and instructor (ROI #2) is estimated by cross-correlation coefficients using a moving window approach (30-s windows and 1-s increments). Positive time lags indicate that the learner leads the BtBC, whereas negative time lags denote that the instructor leads the BtBC. Data shown here are analyzed based on a representative dyad.

We investigated nonverbal behavioral coupling within instructor-learner dyads as a potential mechanism supporting learning and instruction. Beyond previous observations that Body-to-Body Coupling (BtBC) between instructors and learners emerged during learning interactions^11^, we aimed at providing a testbed for whether and how teaching and learning bodies move together in a way that reflects instruction and predicts learning. The instructor was invited to teach psychological concepts to the learner in an ecologically valid situation, during which we used a high-definition digital camera to record behaviors from both learners and instructors simultaneously. A video-based Motion Energy Analysis (MEA) was used to quantify BtBC within dyads^19^ (Fig. 1). MEA is a computer-vision method based on the assessment of differences in sequences of frames in video recordings, requiring no motion capture devices on instructors or learners^19^. MEA along with computer-based algorithms provide a straightforward way to capture and quantify instructor’s and learner’s movements and nonverbal behavioral coordination^20^. Thus, MEA serves as a technique for investigations of learning interactions, particularly appropriate for educators and researchers who wish to record and have access to the pedagogical interaction videos.

In a repeated-measure mixed design, each learner was taught by the instructor with two instructional approaches separated by a brief break (order counterbalanced): (i) once with the *scaffolding* approach (e.g., asking guiding questions or providing hints), and (ii) once with the *explanation* approach (e.g., providing definitions or clarifications). These two approaches were selected because they represent two different influential cognitive engagement activities based on the well-established ‘Interactive-Constructive-Active-Passive’ theory^21^ – constructive engagements embedded in the scaffolding approach and passive engagements in the explanation approach. In addition, considering the importance of adaptive behavior (e.g., personalized guidance) on the part of the instructor in interpersonal learning^22^, each dyad was assigned to one of two groups: (i) Personalized instruction, i.e., the instructor provides personalized guidance based on the informed current level of knowledge of the learner; (ii) Nonpersonalized instruction, i.e., the instructor was not informed in terms of the learner’s current level of knowledge (see Methods for more details).

We hypothesized that BtBC within instructor-learner dyads would be enhanced when the instructor employed a scaffolding strategy compared to an explanation strategy, as the former entails more bidirectional communication and information sharing^23,24^. To the extent that instructor-learner behavioral coupling is pedagogically informative, the enhanced coupling would be reflective of instructional approaches, hypothetically even learning outcomes.

## Results

### Learning outcome

**Fig. 2:**
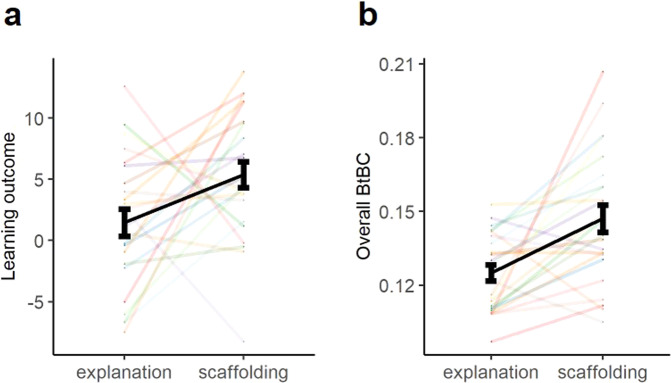
Learning outcome and Body-to-Body Coupling (BtBC). Overall, (**a**) learning outcome (post- minus pre-learning test scores) and (**b**) BtBC for the scaffolding approach was significantly higher than the explanation approach. Colored lines indicate BtBC for each dyad. Black lines denote the averaged BtBC across dyads. Error bars represent standard errors of the mean (s.e.m).

The linear mixed-effects model on learning outcome (i.e., post- minus pre-test score) revealed a main effect of Instructional Approach (*β* = 5.48, SE = 2.20, *t* = 2.48, *p* = 0.02, *R*^*2*^_*β*_ = 0.12), indicating that the learners acquired the concepts better when the instructors used a scaffolding approach (M ± SE, 5.37 ± 1.10) compared to an explanation approach (1.46 ± 1.10) (Fig. 2a). The main effect of Instructional Personalization (*β* = 1.20, SE = 2.20, *t* = 0.54, *p* = 0.59) and the interaction effect (*β* = −3.13, SE = 3.12, *t* = −1.01, *p* = 0.32) were not significant.

### Motion quantity

Before entering dyads’ movement time series into instructor-learner coupling analysis, we tested whether motion quantity was comparable across conditions. The linear mixed-effects model revealed no main effects (*β*s < 0.07, *p*s > 0.18) or interaction (*β* = −0.01, SE = 0.07, *t* = −0.17, *p* = 0.86). A parallel model on the variability (standard deviation) of motion energy did not reveal any significant results either (*β*s < 1.19 * 10^−4^, *p*s > 0.17).

In addition to the dyadic analyses reported above, we explored the individual-level motion quantity. Parallel linear mixed-effects models were performed in the instructors and learners separately. In the instructors, the results showed a main effect of Instructional Approach (*β* = −0.20, SE = 0.07, *t* = −2.68, *p* = 0.01, *R*^*2*^_*β*_ = 0.25), indicating that the instructors moved to a larger degree when using an explanation strategy (0.97 ± 0.06) vs. a scaffolding strategy (0.77 ± 0.06). There was no significant main effect (*β* = 0.21, SE = 0.12, *t* = 1.74, *p* = 0.09) or interaction (*β* = −0.02, SE = 0.10, *t* = −0.19, *p* = 0.85) for Instructional Personalization. Crucially, in the learners, we did not detect any significant effects (*β*s < 0.06, *p*s > 0.19). In sum, no differences in overall motion between conditions were observed for either instructor-learner dyads or learners alone, but instructor motion *did differ* between instructional strategies: Instructors moved more when using an explanation-based instructional approach than when using a scaffolding approach.

### Body-to-Body Coupling

After the motion quantity analysis, motion time series were submitted to the Body-to-Body Coupling (BtBC) analysis. To validate that the observed BtBC did not emerge by chance, we compared overall BtBC (i.e., cross-correlation coefficients averaged over all time lags) from the genuine dataset with the distribution of pseudo-BtBC from the shuffled surrogate dataset. In the combined sample (n = 48), BtBC in genuine dyads (0.14 ± 0.02) was significantly higher than in surrogate dyads (0.08 ± 0.01, permutation test, *p* = 0.0002).

Next, we investigated whether BtBC depended on instructional approaches and personalization. The results from the linear mixed-effects model on overall BtBC showed a main effect of Instructional Approach (*β* = 0.02, SE = 0.01, *t* = 2.25, *p* = 0.03, *R*^*2*^_*β*_ = 0.19; Fig. 2b). Further analysis disclosed that the scaffolding approach (0.15 ± 0.01) was associated with significantly larger BtBC between instructors and learners than the explanation approach (0.13 ± 0.01). No other significant effects were observed (main effect of Instructional Personalization, *β* = 0.01, SE = 0.01, *t* = 0.77, *p* = 0.44; interaction effect, *β* = 0.01, SE = 0.01, *t* = 0.43, *p* = 0.68). The main effect of Instructional Approach remained significant when we added Instructor Motion as an additional fixed factor in the model (*β* = 0.02, SE = 0.01, *t* = 2.29, *p* = 0.03, *R*^*2*^_*β*_ = 0.18).

### Relationship between motion quantity and Body-to-Body Coupling

**Fig. 3:**
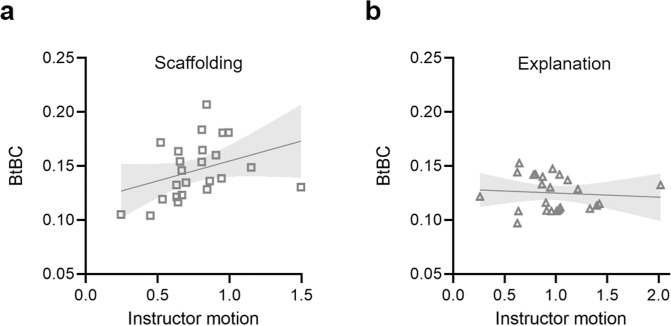
Instructor motion and Body-to-Body Coupling (BtBC). The relationship between instructor motion and BtBC in the scaffolding (**a**) and explanation conditions (**b**).

Based on our observation that instructor movement quantity differed between instructional approaches (scaffolding vs. explanation) and previous studies demonstrating the important role of instructor guidance in learning interaction^25^, we sought to examine whether instructor motion could explain the BtBC effect. To this end, we constructed a linear mixed-effects model on overall BtBC, with Instructional Approach (categorical variable) and Instructor Motion (continuous variable) as two fixed factors and a random intercept of Dyad. This new model generated a significant interaction effect (*β* = 0.04, SE = 0.02, *t* = 2.09, *p* = 0.046, *R*^*2*^_*β*_ = 0.13), indicating that in the scaffolding condition instructor motion was positively associated with BtBC (Fig. 3). Replacing Instructor Motion with Learner Motion in the model did not generate significant effects (*β*s < 0.05, *p*s > 0.06).

### Predicting learning from Body-to-Body Coupling

**Fig. 4:**
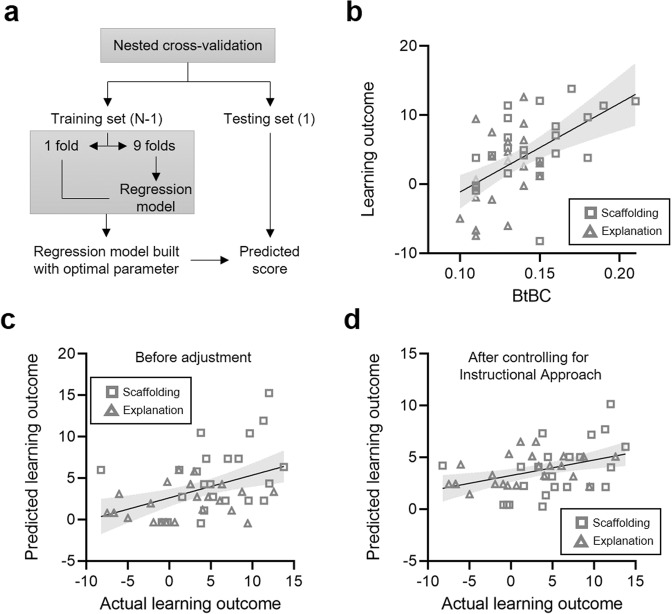
The prediction analyses. (**a**) Nested cross validation (CV) was used for the support vector regression, with the outer leave-one-out CV estimating the generalizability of the model and inner 10-fold CV determining the optimal parameter. (**b**) The linear relationship between body-to-body coupling (BtBC) and learning outcome. (**c**) Raw relationship between actual and predicted learning outcome with combined data of the two instructional approaches. (**d**) Residual relationship between actual and predicted learning outcome after controlling for the effects of scaffolding vs. explanation approaches. Solid line and shared area indicate the linear regression fit and its 95% confidence interval, respectively.

We further tested whether overall BtBC between a learner and an instructor could predict learning outcome (i.e., post- minus pre-test score). Since none of the analyses reported thus far yielded any effect of Personalization, the prediction analysis was applied by combining the two groups (i.e., the personalized and nonpersonalized groups), which had the additional advantage of increasing the power of the analysis. Support vector regression (SVR) analysis with nested cross validation (Fig. 4a) yielded a correlation coefficient of 0.44 (*p* = 0.002, *R*^*2*^ = 0.16) between the actual and predicted learning outcome, indicating that it is possible to infer learning outcome based on overall BtBC alone (Fig. 4b, c). To confirm that BtBC substantially contributed to the learning outcome, we ran an additional prediction analysis after controlling for the effects of instructional approaches (i.e., Instructional Approach as a *covariate* in SVR). The correlation between actual and predicted learning outcome remained significant (*r* = 0.41, *p* = 0.004, *R*^2^ = 0.16, Fig. 4d).

### Decoding instruction from Body-to-Body Coupling

**Fig. 5:**
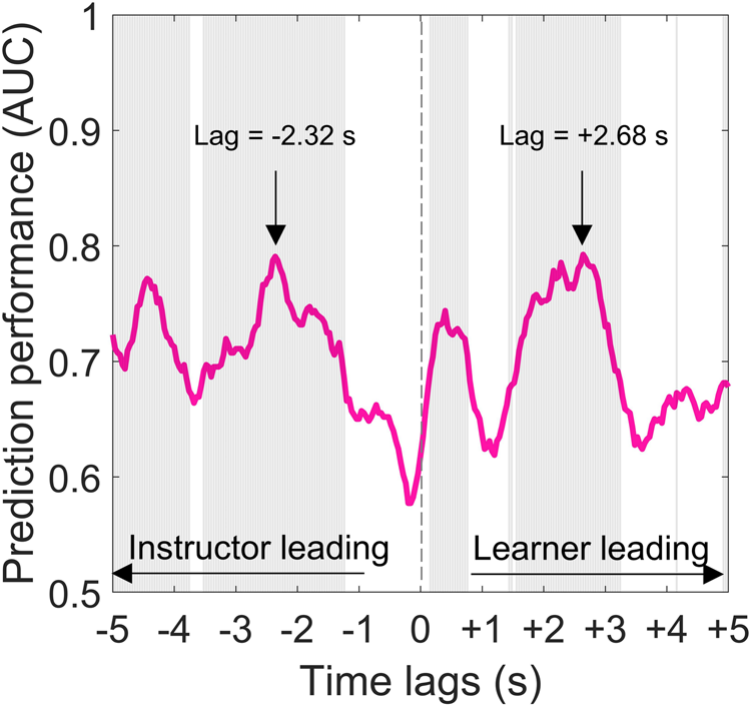
The decoding results. Prediction performance at each time lag, as assessed by area under the receiver operating characteristic curve (AUC). Both instructor leading and learner leading BtBC could successfully decode instructional approaches. Gray regions represent significant prediction performance (FDR corrected *p*s < 0.05). Time lags with the best prediction performance in both directions are labeled.

Finally, we investigated whether the Instructional Approach (scaffolding vs. explanation) employed by an instructor could be decoded from directional leader-follower patterns of BtBC. The logistic regression results showed that both learner leading and instructor leading BtBC could successfully predict the instructional approach (FDR corrected *p*s < 0.05, Fig. 5). In particular, the averaged prediction performance, as assessed by AUC, reached the highest level when the learners’ body motion preceded that of the instructors by 2.68 s (AUC = 0.79, *p* = 6.92 * 10^−4^), and when the instructors’ body motion preceded that of the learners by 2.32 s (AUC = 0.79, *p* = 6.59 * 10^−4^) (Fig. 5). The window of lags between instructors and learners for which BtBC *did* discriminate between instructional strategies, was quite large, dominantly ranging from −5 to −3.72 s and −3.48 to −1.2 s for instructor precedes learner, +0.2 to +0.8 s and +1.48 to +3.28 s for learner precedes instructor. Intriguingly, individual motion energy (averaged within dyads) as the classification feature was insufficient to discriminate between scaffolding and explanation approaches (AUC = 0.62, *p* = 0.13). These results indicate that simultaneous modeling of teaching and learning bodies improves the prediction of instructional approaches over using individual motion alone. Notably, Fig. 5 also clearly shows that the best prediction performance did not occur at lag 0, suggesting that instantaneous instructor-learner body coupling could not provide a more stable prediction performance than the time-lagged data.

### Exploratory analyses

Even though (*i*) the direction of instructor-learner interaction was not the core interest in this study and (*ii*) we did not have an a priori hypothesis for each instructional approach, we first explored the relationship between instructor-/learner-leading BtBC and Instructional Approach in light of elucidating the drivers of BtBC. For learner-leading BtBC (BtBC averaged across all positive lags), we observed a main effect of Instructional Approach (*β* = 0.02, *SE* = 0.01, *t* = 2.11, *p* = 0.047, *R*^*2*^_*β*_ = 0.17), indicating that the scaffolding approach (0.15 ± 0.005) was associated with significantly larger BtBC than the explanation approach (0.13 ± 0.005). The main effect of Personalization (*β* = 0.01, *SE* = 0.01, *t* = 1.08, *p* = 0.28) and interaction effect (*β* = 0.00004, *SE* = 0.01, *t* = 0.03, *p* = 0.98) were not significant. The results suggest that learners might play a more active role in BtBC when instructors used a scaffolding approach (vs. an explanation approach). For instructor-leading BtBC (i.e., BtBC averaged across all negative lags), we observed a trending main effect of Instructional Approach (*β* = 0.02, *SE* = 0.01, *t* = 1.89, *p* = 0.07, *R*^*2*^_*β*_ = 0.14), indicating that the scaffolding approach (0.14 ± 0.005) may be linked to larger BtBC than the explanation approach (0.12 ± 0.005). The main effect of Personalization (*β* = 0.003, *SE* = 0.01, *t* = 0.34, *p* = 0.73) and interaction effect (*β* = 0.01, *SE* = 0.01, *t* = 0.72, *p* = 0.48) were not significant. These results suggest that instructors might also play a more important role in BtBC when using a scaffolding approach than when they use an explanation approach.

Secondly, given that the explanation approach is expected to be more one-directional, we explored the relationship between instructor-leading BtBC and learning outcome in the explanation approach using SVR with nested cross validation (see Methods). However, we did not observe a significant correlation between actual and predicted learning outcome (*r* = 0.17, *p* = 0.42). The results indicate that instructor-leading BtBC was insufficient to predict learning outcome in the explanation approach.

Our third part of exploratory analyses pertains to the null finding of the instructional personalization. To test whether this was due to the instructors failed to adaptively adjust their teaching according to the learners’ knowledge state, we analyzed the relationship between the learners’ pre-test performance and motion quantities (and BtBC). We expected that instructors with successful personalization of instruction would devote more energy (evidenced by the instructors’ entrainment of body movements) to learners with lower prior knowledge. We constructed two linear mixed-effects models:

*Model 1: Motion quantity*_*instructor*_ ~ Pre-test * Personalization + *(1* | *ID)*

*Model 2:* BtBC ~ Pre-test * Personalization + *(1* | *ID)*

The results showed no significant main or interaction effects (Model 1: *β*s < 0.54, *p*s > 0.14; Model 2: *β*s < 0.05, *p*s > 0.17), suggesting that the instructors might have failed to adaptively adjust their teaching based on the learners’ knowledge state.

## Discussion

Body movement is a fundamental nonverbal vehicle of social interaction in the human species: the coordination of body movements is crucial for bidirectional communication and information sharing in a social world. In this study, we sought to characterize dyadic body movements during naturalistic instructor-learner interactions. Using a video-based Motion Energy approach, we investigated Body-to-Body Coupling (BtBC) within instructor-learner dyads and its relationship with instruction and learning (i.e., instructional approaches and learning outcome). We made several key observations in this study.

First, we found similar dyad movement quantities across groups and conditions. We did not observe a significant difference in average dyad motion energy between scaffolding and explanation conditions, indicating that dyadic movement quantity alone does not reflect the functional differences in these instructional approaches despite their differences in terms of instructional goals and strategies^26^. When we zoomed in and tested the motion quantity at the individual level, however, we *did* find that instructor motion varied between instructional strategies: overall, instructors moved more when using an explanation approach compared to scaffolding. As such, these results extend previous studies where the movement was identified as a pedagogic tool in teaching^27^ and reported to facilitate learning^28,29^, to scaffolding vs. explanation approaches. To date, there has been scarcely any empirical research comparing the nonverbal behaviors between these two approaches.

Body-to-body coupling (BtBC; i.e., coordinated movements between instructors and learners) also depended on the instructional approach. Specifically, we observed stronger BtBC within dyads when the instructor used a scaffolding approach compared to an explanation approach, just like we previously reported for inter-brain coupling during learning interactions^30^. This finding is further in line with prior work suggesting that BtBC reflects real-time interpersonal information sharing^8^ and holds relevance for empathy^31^ and affiliation^7^.

Contrary to our expectation, we did not find an effect of instructional personalization (i.e., adaptive instruction^22^) on learning and BtBC. The absence of effects might be interpreted in terms of the following two possible scenarios: (i) the instructors failed to adaptively adjust their teaching according to the learners’ knowledge state, or (ii) the learners were unresponsive to such personalization (see also previous research for challenges on instructional personalization^32^). To arbitrate between these possibilities, we analyzed the relationship between the learners’ pretest performance and instructor motion quantities (and BtBC). We expected that instructors with successful personalization of instruction would devote more energy (evidenced by the instructors’ entrainment of body movements) to learners with lower prior knowledge (see the Exploratory Analyses section). The linear mixed-effects models revealed no significant effects, suggesting that the instructors might have failed to adaptively adjust their teaching based on the learners’ knowledge state. It is important to note that our results do not necessarily invalidate the effectiveness of instructional personalization, as it is possible that personalization cannot be captured in terms of body movement. The precise role of personalization in BtBC is of great interest and requires further research.

**Fig. 6:**
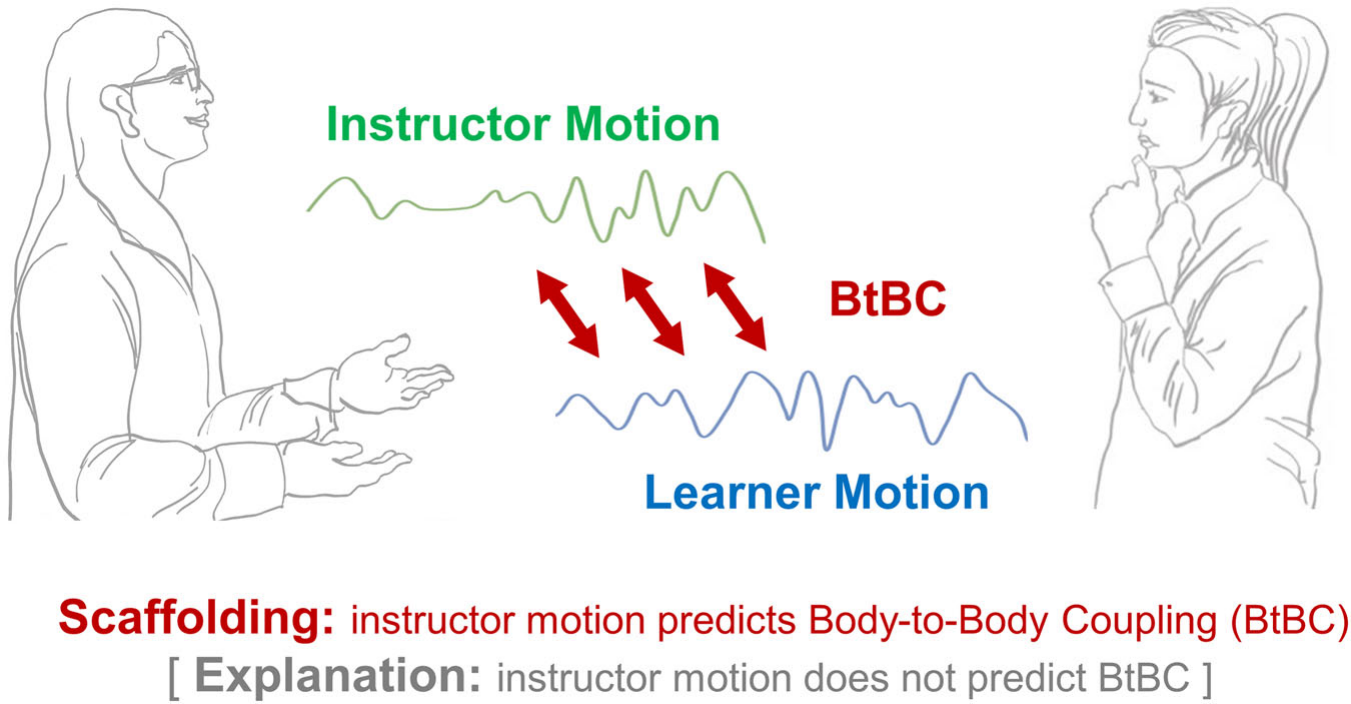
A synthesized framework for instructor motion quantity and Body-to-Body Coupling (BtBC). During learning interaction, the scaffolding strategy triggers nonverbal motion in the instructors that might entrain motion in the learners, coordinating body sway within instructor-learner dyads. In the scaffolding condition, as instructor motion increased, BtBC enhanced accordingly. In the explanation condition, such a positive relationship was absent.

Crucially, there was a positive relationship between instructor motion and BtBC, but only in the scaffolding condition. One interpretation for this finding is that instructor motion as nonverbal guidance might help entrain the learner motion, coordinating body sway between instructor and learner (i.e., BtBC), which could further support effective learning (Fig. 5). But this mechanistic feature depended on the instructional approach: it worked for scaffolding. When the instructors used an explanation approach, their motion quantity did not contribute to BtBC. Notably, in general, instructors moved even more during explanation interactions (vs. scaffolding interactions), which implies that the frequency of movement itself did not necessarily lead to BtBC. Rather, *how* instructors swayed might be the secret to successfully coordinated body movements (Fig. 6).

Our observations are compatible with the ‘Interactive-Constructive-Active-Passive’ theory^21^. With scaffolding, the instructor had to provide the learner with hints and guiding questions, which can help the learner regulate their learning and integrate new information with prior knowledge^23,26^. This process requires constructive engagement in the learning activities and might therefore increase verbal and nonverbal bidirectional communications and coordination between instructor and learner. In contrast, with explanation, learners often passively receive concepts and clarification from the instructor, leading to unidirectional communication and constrained engagement in elaborative activities^26^. In support of this model, we were able to infer learning outcomes based on BtBC. This finding replicates our recent work where spontaneous and synchronized movements between instructor and learner predicted intonation learning performance during the acquisition of music songs^20^. An important next question is whether BtBC may predict not only immediate learning outcomes but also long-term memory retention.

Finally, BtBC successfully distinguished between instructional approaches. Apart from confirming that bidirectional communication between instructor and learner contains information that classifies instruction, this finding additionally hint at a potential directionality in BtBC. Indeed, neurophysiological studies in the field of instructor-learner interaction suggest that before the knowledge is actually transmitted, instructors *predict* the learner’s knowledge state. Learners, in turn, imitate the demonstration delivered by the instructors^33,34^. This leads to the hypothesis that the instructor leads the learning interaction. Importantly, however, instructors are also tasked with providing guidance to learners. To do so, they might also have to *follow* the learners’ knowledge state and adjust their instruction or timing of demonstration to the learners’ progress accordingly^34^; if this is true, we would expect learners to lead the learning interaction as well on some occasions. In this study, we provide evidence in support of such bidirectionality. As such, our findings can be conceptualized in the context of a mutual adaptation system^35^: In some instances during the dynamic learning process, instructors adapted to learners, as revealed by the difference in correlations at negative lags; in other instances, learners adapted to instructors, as revealed by the difference in correlations at positive lags. In other words, BtBC between learners and instructors was achieved and maintained via continuous mutual adaptation (lagged correlation). It is this mutual adaptation, and not perfect synchronization (zero-lag correlation), that supports the dynamic learning process. Our findings are compatible with the view that successful learning might be associated with a balance between learners leading vs. instructors leading^36^.

This study has some limitations, one of which is the sample size. This is a common issue in dyadic research and limits statistical power^37^. We used both logistic regression and support vector regression to decode instruction and learning from BtBC respectively, providing valuable demonstrations for the application of machine learning in pedagogical studies. Albeit all of these methods have merits and have been successfully applied in previous multiperson studies^30,38–40^, the findings derived from this study require future replications based on larger sample size. A second important weakness pertains to the clear gender bias. We only tested female-female learning interactions. This practice was aimed at controlling the effect of gender composition on social behaviors^41^. Future studies should consolidate the current findings in both same-gender and mixed-gender dyads. Another limitation concerns the MEA method itself. This is an easy-to-use approach to quantify the synchrony between individuals. However, given the fact that the pre-defined ROIs are fixed, MEA cannot track moving ROIs and movement details. It would be ideal in the future to combine MEA with other motion capture devices or approaches (such as OpenPose^42^) that detect the human body, hand, facial, and foot joints. This practice would provide more information about *how* instructors (and learners) moved when using a certain instruction.

The co-occurrence of body-to-body coupling (BtBC) and learning raises critical questions regarding causality. Is BtBC functionally contributing to learning, or does BtBC simply emerge as a byproduct of learning? The evidence presented here is correlational and cannot be used to make causal inferences. Future studies could exogenously manipulate BtBC by using e.g., synchronous rhythmic arm movement^43^ and observe its effect on subsequent learning interaction and outcome.

Finally, future research is needed to combine BtBC and instructor-learner interactions to study e.g., learning difficulties. Recent years have witnessed fruitful applications of BtBC in clinical areas; for example, coordinated body movements reflected relationship quality and outcome in psychotherapy^9^, and were closely related to symptom profiles of patients with Schizophrenia^44^ and Borderline Personality Pathology^45^. The degree of BtBC may be indicative of core deficits underlying interpersonal communication and social functioning (including learning interactions). Thus, BtBC can serve as an objective and sensitive measure, providing new insights into relationships between learning outcome, behaviors, and communicative deficits in underachievers.

In sum, this study contributes to our understanding of pedagogical interactions in (at least) two aspects: teaching and learning bodies move together during interactions; such BtBC within instructor-learner dyads predicts learning outcome and reflects instructional approaches, underscoring its functional significance. Methodological advances open unprecedented opportunities to study how coordinated movements and behaviors during interaction help learning.

## Methods

### Participants

Forty-eight female, healthy, right-handed adults were recruited through advertisements spread within East China Normal University. Half of the participants (*n* = 24, age: M ± SD, 22.58 ± 2.75 years) were recruited as instructors. They majored in psychology, had received training as a teacher for at least 1 year, and were familiar with the learning content. The other 24 participants (age: 20.33 ± 2.30 years), who majored in non-psychology-related fields and had not been exposed to the learning content, were recruited as learners. The instructors and learners were not acquainted. We recruited same-gender dyads to control for potential gender effects on interactions^41^. All participants received monetary compensation for their participation and provided signed written informed consent before the experiment. The study procedure was approved by the University Committee of Human Research Protection (HR 044-2017), East China Normal University.

### Experimental procedures and materials

The instructors and the learners were invited to complete a conceptual learning task. The task was composed of two blocks, each entailing a 3-min rest phase and an 8-min learning phase. During the rest phase, both participants in a dyad were asked to relax and no communication was allowed. During the learning phase, the instructor and the learner engaged in interactive learning. We manipulated two factors during the experiment: (i) Instructional Approach (scaffolding vs. explanation, within-dyad factor) and (*ii*) Instructional Personalization (personalized vs. nonpersonalized, between-dyad factor). For each task block, the instructor would either guide the learner in a Q&A manner (scaffolding) or explain the concept to the learner and provide examples (explanation). Examples for scaffolding include asking guiding questions or providing hints; examples for explanation include providing definitions or clarifications. Half of the participants (*n* = 12 dyads) were assigned to the personalized group: the instructor was provided with the learner’s pretest scores (so they were able to know the learner’s lack of understanding) and was asked to customize their instructions to the learner’s aptitude and ability. For example, the instructor could adjust the teaching of certain concepts if the learner was unclear about them as established via the pretest. For the other non-personalized group (*n* = 12 dyads), no information about the learner’s prior knowledge was provided for the instructor.

Learning materials included two sets of psychological concepts: *reinforcement* (e.g., positive reinforcement, negative reinforcement, punishment, and retreat) and *transfer* (e.g., near transfer, far transfer, lateral transfer, and vertical transfer), chosen from a national standard textbook (Educational Psychology: A Book for Teachers). These materials were proven to be of reasonable test reliability (Cronbach’s *a* = 0.81) and content validity. For each block, the instructor was asked to teach one of the two sets of concepts to the learner. The learners in our sample had never been exposed to this particular learning content. The learning content (i.e., “reinforcement” and “transfer”) was randomly assigned to the two instructional approaches. For each group, the experimental procedure consisted of one of the following two combinations of learning content and instructional approaches: (*i*) Block 1: reinforcement + scaffolding, Block 2: transfer + explanation; (*ii*) Block 1: reinforcement + explanation, Block 2: transfer + scaffolding. Block order was counterbalanced.

Two days prior to the formal experiment, all instructors were informed and trained by experimenters. The instructors underwent standardized training to ensure that they fully understood the teaching materials, which entailed instructional goals, instructional difficulties, general instructional processes, and detailed instructional scripts (see Supplementary Table 1 for an example). These instructional scripts were created by two psychological experts (>20 years of instructional experience at the university level), aiming at maintaining the consistency of the number of questions and the concepts across all the participant dyads. The instructors rehearsed the teaching material based on the script, to fit it within 8 minutes. They practiced instruction with the experimenter one-on-one and only proceeded to the experiment phase once their performance met the established standardization requirements. Teaching performance was operationalized in terms of the following aspects: the length of teaching, speech rate, and consistency with the instructional processes and scripts^30,46^. At the beginning of the teaching script for the scaffolding approach, some basic knowledge on the topic was given (but this was minimally delivered, to maintain a clear distinction from the explanation approach). The teaching script was designed for an 8-min long learning phase, but in case there was remaining time, the instructors were asked to repeat the same information until the end of the phase (this happened in 3 dyads only, and only for about 60 seconds).

To confirm that the two approaches used the given 8-min learning phase equally in an optimal manner for each approach, we performed a video coding analysis. Each 1-s video fragment from the 8-min learning phase was coded as either containing verbal interactions or not in both conditions (see our recent work^30^ for the detailed video-coding procedure). To confirm that the two approaches used the given 8-min learning phase equally in an optimal manner, we reported the average talking duration by the instructors and learners (p. xx). Specifically, we computed the duration ratio of talking by quantifying the proportions of time (out of 8 min) when verbal interactions occurred. For example, if the whole verbal interaction including all turn-takings occurred for a total of 7 min (1-min silent thinking or nonverbal interaction for the reminder of the time), then the duration ratio of talking should be 7/8 = 0.875. We found that though instructors in the explanation condition talked more than those in the scaffolding condition (0.77 ± 0.13 vs. 0.56 ± 0.18, *t*_11_ = 3.18, *p* = 0.009), the total talk-ratio of instructor-learner dyads was comparable between scaffolding and explanation conditions (0.91 ± 0.04 vs. 0.90 ± 0.10, *t*_11_ = 0.39, *p* = 0.70).

During the whole experiment, participants’ body movements were recorded using a digital video camera (Sony, HDR-XR100, Sony Corporation, Tokyo, Japan), with approximately 90° angle in between the shooting perspective and chairs’ orientations. The instructor and the learner’s chairs were slightly oriented towards the camera to improve whole-body visibility (Fig. 1a). Their brain activity was recorded simultaneously via functional near-infrared spectroscopy; brain imaging results are reported elsewhere^30^.

### Learning assessment

The learning outcome was evaluated before the onset of the rest phase and after the end of the learning phase (i.e., post- minus pretest score). A total of 8 definitions, 16 true-false items, and 4 simple answer questions were selected from the textbook (as above) as the test bank. Half of the items were used for the pre-learning test and half for the postlearning test. The learning outcome was indexed by the difference between the pre- and postlearning test scores. Importantly, pretest scores did not differ between conditions and groups (*t*s < 2.07, *p*s > 0.06).

### Motion energy analysis

Analysis of the video recordings (MP4 format, dimension 1920 * 1080, FPS 25, 12000 frames), was focused on the 8-min learning phase, as this phase involved interactions between instructors and learners. Body-to-Body Coupling (BtBC) between instructors and learners was estimated using a motion energy analysis (MEA) algorithm^19^. MEA is an objective frame-differencing method to quantify the pixel changes in movement from videos, i.e., motion energy^9,19^. The algorithm automatedly monitors and computes the number of pixels changing across video frames within predefined regions of interest (ROIs). The motion energy analysis entailed the following four steps^19,20^. First, the MEA algorithm was applied to two separate ROIs: ROI #1 for the whole body of the instructor and ROI #2 for that of the learner (Fig. 1a). A continuous measure of movement associated with either the instructor or the learner was computed and extracted (Fig. 1b, c). Second, extracted raw motion time series for both instructor and learner were preprocessed: signals were smoothed using a moving average window (window size = 0.5 s); values exceeding mean plus 10 * standard deviations of the time series were identified as outliers and removed (<0.2% of the whole data); the time series were then standardized. Third, the preprocessed data were submitted to a cross-correlation analysis. Specifically, for each dyad and condition, motion energies associated with the instructor and the learner were cross-correlated (Fig. 1d). This was done based on moving windows (window size = 30 s), which is appropriate given the non-stationary nature of movement behaviors^47^. The maximum time lag was set to 5 s (i.e., ±5s, in both directions) in steps of 0.04 s, resulting in 125 steps in each direction. These parameters are in accordance with previous studies^9,20^, which makes the analyses maximally comparable. Finally, BtBC was estimated using window-averaged, Fisher’s z transformed, absolute cross-correlation coefficients, which comprised 251 time lags (125 positive lags, 125 negative lags, and the zero lag). Consequently, both positive and negative cross-correlation coefficients contributed positively to BtBC. In line with previous work^9,44,45^, we derived an index of overall BtBC, i.e., cross-correlation coefficients averaged over all time lags. To control for spurious BtBC, we created a surrogate dataset (*n* = 100 out of each genuine time series) by segment-wise (60-s segments) shuffling of the original data^9,19^. For example, the first minute of the instructor’s time series was re-paired with the third minute of the learner’s time series. This within-dyad shuffling procedure aligned instructor’s and learner’s movement segments that never occurred at the same time (i.e., pseudo-interactions), while keeping the time structure of data intact. Data preprocessing and MEA were implemented using the rMEA package^48^ and custom-made codes in R (statistical environment, version 3.6.3; R Core Team, 2020).

### Statistical tests

For statistical analysis, we used linear mixed-effects models. The models were constructed using the *lme4* package in R^49^. Significance tests on the parameters yielded by the model were performed using the *lmerTest* package in R^50^. The *R*^*2*^_*β*_ statistics were estimated to describe goodness-of-fit for the linear mixed-effects models using the Kenward-Roger approach implemented in the *r2glmm* package in R^51^. For significant (thresholded at *p* < 0.05, two-sided) main effects or interaction, follow-up contrasts were conducted using the *emmeans* package in R^52^.

#### Learning outcome

The dependent variable was post- minus pretest score. Data were modeled by Instructional Approach (scaffolding vs. explanations), Instructional Personalization (personalized vs. non-personalized), and their interaction. The random intercept of Dyad was taken into account.

#### Motion quantity

The outcome variable was the individual motion energy of each person in each dyad for each condition. Data were modeled by Instructional Approach (scaffolding vs. explanation), Instructional Personalization (personalized vs. non-personalized), and the interaction of these fixed effects. Random intercepts were estimated for Participant and Dyad, with the former being nested within the latter.

#### Body-to-Body Coupling (BtBC)

BtBC was estimated using MEA. Two complementary analyses were then conducted on BtBC. First, we aimed at determining that the observed BtBC was specific to real dyads. The genuine BtBC from the real dyads were compared with the distribution of pseudo-BtBC derived from the shuffled surrogate dataset using a permutation test (thresholded at *p* < 0.05). Second, we evaluated instruction-related BtBC, i.e., whether BtBC depended on instructional approaches and/or personalization. To this end, Instructional Approach (scaffolding vs. explanation), Instructional Personalization (personalized vs. non-personalized), and the interaction of these effects was tested for overall BtBC. Data were analyzed using the same multilevel modeling above, with Dyad as a random intercept.

#### Predicting learning from BtBC

We tested whether it is possible to infer learning outcomes based on overall BtBC (predictor). To this end, we used linear support vector regression (SVR). A nested cross-validation (CV) approach was used, with the outer leave-one-out CV estimating the generalizability of the model and inner 10-fold CV determining the optimal parameter (Fig. 4a). Prediction performance of regression was expressed by the Pearson correlation coefficient between predicted and actual response values^53,54^. The coefficient of determination (denoted by *R*^*2*^) was also reported. To confirm that BtBC had substantial contribution to the learning outcome, we re-run the prediction analysis after controlling for the effects of instructional approaches (i.e., Instructional Approach as a *covariate* in SVR), as has been conducted in a recent paper^55^. SVR were constructed using standard functions and custom codes in MATLAB 2019b.

#### Decoding instruction from BtBC

To test BtBC’s ability to classify the different instructional approaches, the prediction performance of BtBC was estimated using the area under the curve (AUC) of receiver operating characteristic (ROC). We used logistic regression as the classification algorithm^30,53^. The logistic regression-based decoding analysis allows us to quantify how well scaffolding and explanation approaches (classification label) can be distinguished by BtBC (classification feature). To evaluate the significance of AUC, we performed a series of permutation analyses. The chance level of the AUC was estimated by randomly shuffling the labels for BtBC (i.e., window-averaged, Fisher-z transformed, absolute cross-correlation coefficients) to produce a null distribution. For example, BtBC for the first dyad in the scaffolding condition can be re-labeled with “explanation” or “scaffolding” (50% probability) through the shuffling. Significance levels (thresholded at *p* < 0.05) were determined by comparing the AUC from the real labels with 1000 renditions of randomized labels. This permutation analysis was repeated for each time lag (i.e., 251 times). Benjamini-Hochberg False-Discovery-Rate (FDR) method was used to correct for multiple comparisons^56^. Logistic regression models were constructed using standard functions and custom codes in MATLAB 2019b.

### Reporting summary

Further information on research design is available in the Nature Research Reporting Summary linked to this article.

## Supplementary information

Supplemental Material

Reporting Summary

## Data Availability

The data supporting the main findings of this manuscript are available from the OSF repository (https://osf.io/49mga/) and the corresponding authors upon reasonable request. Motion Energy Analysis (MEA) and statistical analyses were conducted with standard toolboxes in R 3.6.3. Regression models were constructed using standard functions and custom codes in MATLAB 2019b. Further inquiries can be directed to the corresponding authors.
