# Supplementary material for: Instructor-learner body coupling reflects instruction and learning
Source: NPJ Sci Learn. 2022 Jun 28;7:15. doi: 10.1038/s41539-022-00131-0 (PMC9240028; doi:10.1038/s41539-022-00131-0)
Supplement: Supplementary file 1 — Supplemental Material [file 41539_2022_131_MOESM1_ESM.pdf]

Supplementary Materials

Supplementary Table 1. Example teaching scripts (translated from Chinese)

	Scripts
Scaffolding	Instructor: Positive reinforcement refers to rewarding goal-directed behavior to increase its frequency. How can one provide positive reinforcement?
	Learner: …By rewarding positive behavior?
	Instructor: Bingo! Could you please give an example?
	Learner: My sister gave me some candies after I cleaned my room.
	……
Explanation	Instructor: Positive reinforcement refers to rewarding goal-directed behavior to increase its frequency. Do you see what I mean?
	Learner: I am not sure whether I understand it correctly. Could you please explain it a bit more?
	Instructor: For example, my mom cooks my favorite food for me when I pass exams.
	Learner: That clarifies it.
	……
