# Supplementary material for: Instructor-learner body coupling reflects instruction and learning
Source: NPJ Sci Learn. 2022 Jun 28;7:15. doi: 10.1038/s41539-022-00131-0 (PMC9240028; doi:10.1038/s41539-022-00131-0)
Supplement: Supplementary file 2 — Reporting Summary [file 41539_2022_131_MOESM2_ESM.pdf]

nature portfolio

Corresponding author(s): Pavel Goldstein, Yi Hu

Last updated by author(s): Mar 30, 2022

Reporting Summary

Nature Portfolio wishes to improve the reproducibility of the work that we publish. This form provides structure for consistency and transparency in reporting. For further information on Nature Portfolio policies, see our Editorial Policies and the Editorial Policy Checklist.

Statistics

For all statistical analyses, confirm that the following items are present in the figure legend, table legend, main text, or Methods section.

n/a	Confirmed	
☐	☒	The exact sample size (n) for each experimental group/condition, given as a discrete number and unit of measurement
☐	☒	A statement on whether measurements were taken from distinct samples or whether the same sample was measured repeatedly
☐	☒	The statistical test(s) used AND whether they are one- or two-sided<br>*Only common tests should be described solely by name; describe more complex techniques in the Methods section.*
☐	☒	A description of all covariates tested
☐	☒	A description of any assumptions or corrections, such as tests of normality and adjustment for multiple comparisons
☐	☒	A full description of the statistical parameters including central tendency (e.g. means) or other basic estimates (e.g. regression coefficient) AND variation (e.g. standard deviation) or associated estimates of uncertainty (e.g. confidence intervals)
☐	☒	For null hypothesis testing, the test statistic (e.g. F, t, r) with confidence intervals, effect sizes, degrees of freedom and P value noted<br>*Give P values as exact values whenever suitable.*
☒	☐	For Bayesian analysis, information on the choice of priors and Markov chain Monte Carlo settings
☒	☐	For hierarchical and complex designs, identification of the appropriate level for tests and full reporting of outcomes
☐	☒	Estimates of effect sizes (e.g. Cohen's d, Pearson's r), indicating how they were calculated

Our web collection on statistics for biologists contains articles on many of the points above.

Software and code

Policy information about availability of computer code

Data collection	N/A
Data analysis	Motion Energy Analysis (MEA) and statistical analyses were conducted with standard toolboxes in R 3.6.3. Regression models were constructed using standard functions and custom codes in MATLAB 2019b.

For manuscripts utilizing custom algorithms or software that are central to the research but not yet described in published literature, software must be made available to editors and reviewers. We strongly encourage code deposition in a community repository (e.g. GitHub). See the Nature Portfolio guidelines for submitting code & software for further information.

Data

Policy information about availability of data

All manuscripts must include a data availability statement. This statement should provide the following information, where applicable:

- Accession codes, unique identifiers, or web links for publicly available datasets
- A description of any restrictions on data availability
- For clinical datasets or third party data, please ensure that the statement adheres to our policy

The data supporting the main findings of this manuscript are available from the OSF repository (https://osf.io/49mga/) and the corresponding authors upon reasonable request. Motion Energy Analysis (MEA) and statistical analyses were conducted with standard toolboxes in R 3.6.3. Regression models were constructed using standard functions and custom codes in MATLAB 2019b. Further inquiries can be directed to the corresponding authors.

Field-specific reporting

Please select the one below that is the best fit for your research. If you are not sure, read the appropriate sections before making your selection.

☐ Life sciences ☒ Behavioural & social sciences ☐ Ecological, evolutionary & environmental sciences

For a reference copy of the document with all sections, see nature.com/documents/nr-reporting-summary-flat.pdf

Behavioural & social sciences study design

All studies must disclose on these points even when the disclosure is negative.

Study description	Quantitative experimental study
Research sample	Forty-eight female, healthy, right-handed adults were recruited through advertisements spread within East China Normal University. Half of the participants (n = 24, age: M ± SD, 22.58 ± 2.75 years) were recruited as instructors. They majored in psychology, had received training as a teacher for at least 1 year, and were familiar with the learning content. The other 24 participants (age: 20.33 ± 2.30 years), who majored in non-psychology-related fields and had not been exposed to the learning content, were recruited as learners. The instructors and learners were not acquainted. We recruited same-gender dyads to control for potential gender effects on interactions.
Sampling strategy	Random sampling. No statistical methods were used to predetermine sample size, but our sample size is similar to those reported in previous studies using the instructor-learner interaction paradigm (e.g., Bevilacqua et al., 2019; Pan et al., 2018; Takeuchi et al., 2017) as well as studies conducting prediction analyses based on two-person synchrony data (e.g., Dai et al., 2018; Jiang et al., 2012).
Data collection	During the whole experiment, participants' body movements were recorded using a digital video camera (Sony, HDR-XR100, Sony Corporation, Tokyo, Japan), with approximately 90° angle in between the shooting perspective and chairs' orientations. The instructor and the learner's chairs were slightly oriented towards the camera to improve whole-body visibility. No one else was present besides the participants and the researcher. The researcher was blind to the study hypothesis during data collection.
Timing	From July 2017 to December 2017
Data exclusions	Raw motion time series for both instructor and learner were preprocessed: values exceeding mean plus 10 * standard deviations of the time series were identified as outliers and removed (< 0.2% of the whole data).
Non-participation	No participants declined participation.
Randomization	Participants were randomly allocated to each experimental group.

Reporting for specific materials, systems and methods

We require information from authors about some types of materials, experimental systems and methods used in many studies. Here, indicate whether each material, system or method listed is relevant to your study. If you are not sure if a list item applies to your research, read the appropriate section before selecting a response.

Materials & experimental systems

n/a	Involved in the study	
☒	☐	Antibodies
☒	☐	Eukaryotic cell lines
☒	☐	Palaeontology and archaeology
☒	☐	Animals and other organisms
☐	☒	Human research participants
☒	☐	Clinical data
☒	☐	Dual use research of concern

Methods

n/a	Involved in the study	
☒	☐	ChIP-seq
☒	☐	Flow cytometry
☒	☐	MRI-based neuroimaging

Human research participants

Policy information about studies involving human research participants

Population characteristics	See above.
Recruitment	Participants were recruited through advertisements spread within East China Normal University.
Ethics oversight	University Committee of Human Research Protection (HR 044-2017), East China Normal University

Note that full information on the approval of the study protocol must also be provided in the manuscript.
